# Gender differences and typical nutrition concerns of the diets of preschool children – the results of the first stage of an intervention study

**DOI:** 10.1186/s12887-017-0962-1

**Published:** 2017-12-19

**Authors:** Sylwia Merkiel-Pawłowska, Wojciech Chalcarz

**Affiliations:** Food and Nutrition Department of the Eugeniusz Piasecki University School of Physical Education in Poznan, Królowej Jadwigi 27/39 Street, 61-871 Poznan, Poland

**Keywords:** Preschool children, Nutrient intake, Energy, Macronutrients, Vitamins, Minerals, Intervention, Nutrition, Diet

## Abstract

**Background:**

Nutrition in children has an important influence on health both in childhood and adulthood. Actions aimed at improving children’s nutrition are essential, not only to the children and their families, but also to the whole society. The aim of the study was to present the results of nutrient intake before starting a nutrition and physical activity intervention programme, to investigate gender differences in nutrient intake and to discuss whether the preschoolers’ nutrient intake is similar to the intake of their peers from other countries.

**Methods:**

Nutrient intake was estimated from seven-day weighed food records kept by parents and preschool staff individually for 122 4–6-year-old children who attended two preschools in Piła, north-western Poland. Nutrient intake was calculated using Dieta 4.0 computer programme including water intake and intake of nutrients from dietary supplements. Statistical analysis was performed using the IBM SPSS Statistics computer programme, version 21.0, according to gender. The study was approved by the Bioethics Committee of the Poznan University of Medical Sciences (reference number 886/08).

**Results:**

Boys, in comparison to girls, were found to have statistically significantly higher intakes of total protein, total protein per kg of body weight, saturated fatty acids, total carbohydrates, available carbohydrates, lactose, sucrose, total water, vitamin A, beta-carotene, vitamin B_2_, vitamin B_12_, vitamin C, calcium, phosphorus, and magnesium. Statistically significantly higher percentage of girls than boys had intakes of vitamin E below AI.

**Conclusions:**

Gender was a significant factor of nutrient intake in the studied preschool children. The main nutritional concerns in the studied preschoolers’ diets, irrespective of gender, are typical of the diets of preschool children from various parts of Europe and indicate the need to work out common nutritional strategies to improve preschoolers’ nutrition across Europe to reduce future burden of diet-related diseases to the European societies.

## Background

Nutrition plays a crucial role in human growth, development and health [1]. Adequate nutrition during childhood not only has an important influence on the current health status of the child but also prevents diet-related diseases in adulthood [1, 2]. Nevertheless, studies have showed many dietary inadequacies in children of all ages [e.g. [3–7]]. These inadequacies are reflected in biochemical blood indices [e.g. [3, 8]] and inadequate hydration status [5] all of which may have adverse effect on health, both at present and in the future. A diet modelling study based on NHANES 2007–2010 [9] showed that consuming the daily recommended amounts of dairy products would reduce the prevalence of inadequate micronutrient intakes in children of all ages. This study proved that dietary interventions are necessary to improve children’s nutrition. Planning and carrying out such interventions is essential not only to the children themselves and to their families, due to increasing the quality of life and the possibilities of personal physical, mental and emotional development [1], but also to the whole society, due to reducing the costs of health care, reducing absence rate at work due to illness and increasing the productivity [10–12].

To plan a nutrition intervention adequately, it is necessary to know which nutrients should be targeted to successfully balance children’s diets. The results should be analysed separately for girls and boys because the effect of an intervention may be influenced by gender [13]. Moreover, gender should be taken into account because this variable was showed to have different influence on the response to diet [14–16]. Although this difference was showed in adults, tracking dietary behaviour [17–19] indicates that as early as at preschool age children should be taught such dietary behaviour which is expected in older childhood, adolescence and adulthood.

Many nutrition intervention programmes for preschool children have been presented in the international literature so far [20]. However, to our knowledge, we carried out the first nutrition intervention programme for Polish preschool children, which aimed to modify diet and physical activity in preschool children in order to improve their nutritional status and physical fitness. In the current article, we report the results of nutrient intake from the first stage of the intervention programme, that is before introducing education of the preschool staff and parents, and before modifying children’s diets. Apart from presenting the results, our aim was also to investigate any potential gender differences in nutrient intake and to discuss whether the studied preschoolers’ nutrient intake is similar to the intake of their peers from other countries based on the published literature.

## Methods

### General information

The outline and detailed methodology of the whole intervention programme were presented in the previous articles [21, 22]. The study was approved by the Bioethics Committee of the Poznan University of Medical Sciences. Only children whose parents provided written consent took part in the study.

### Subjects

From among 234 children who attended two randomly selected preschools in Piła, north-western Poland, complete and valid food records were provided by parents of 122 4–6-year-old children. All the details on the subjects exclusion were provided in the previous article [23].

### Nutrient intake

#### Data collection

Nutrient intake was estimated from seven-day weighed food records kept by parents and preschool staff individually for each child, including water and any dietary supplements. Both parents and preschool staff were instructed how to keep the food record, how to weigh foods and beverages, especially the ingredients of complex dishes, and how to weigh leftovers [22]. They were equipped with Soehnle Page 66100 electronic scales. The authors helped the staff in keeping the food records at preschools and were fully available (both personally at preschools and on the telephone) to parents in case help was needed. All the details on data collection were provided previously [22, 23].

#### Dietary assessment and comparison with nutritional guidelines

Two underreporters were identified and excluded from further analysis as described previously [23]. Intakes of macronutrients, vitamins and minerals were calculated using Dieta 4.0 computer programme and modified as described previously [21] for the sake of the data accuracy. The Microsoft Excel 2010 computer programme was used to calculate intake of total protein per kg of body weight, and intake of animal and plant protein expressed as % of total protein.

Intakes of nutrients were compared to the Dietary Reference Values (DRVs) for the Polish population [24], that is: (1) to the Estimated Average Requirement (EAR) in the case of total protein per kg of body weight; vitamins A, B_1_, B_2_, B_6_, B_12_, C, folic acid and niacin; calcium, phosphorus, magnesium, iron, zinc, copper and iodine; (2) to the Recommended Dietary Allowance (RDA) in the case of available carbohydrates; (3) to the Adequate Intake (AI) in the case of dietary fibre, total water, vitamin E, sodium, potassium; (4) and to the Tolerable Upper Intake Level (UL) in the case of sodium. Since the Polish DRVs include only AI for vitamin D intake and do not include manganese at all, the DRVs worked out by the Food and Nutrition Board of the Institute of Medicine were used, that is EAR in the case of vitamin D [25] and AI in the case of manganese [26]. Moreover, the Polish DRVs include UL only for sodium, therefore, the ULs worked out by the Scientific Committee on Food [27] were used for retinol, vitamin D, E, B_6_, folate, zinc, copper and iodine, and the ULs worked out by the Food and Nutrition Board of the Institute of Medicine [25, 26, 28–30] were used for niacin, vitamin C, calcium, phosphorus, iron and manganese. We also compared cholesterol intake to the intake recommended by the World Health Organization in the prevention of diet-related diseases [31].

#### Statistical analysis

Statistical analysis was performed using the IBM SPSS Statistics computer programme, version 21.0 (Armonk, NY: IBM Corp.). All the results were analysed according to gender. Means and standard deviations (SD) were calculated. However, since in the case of AI assessing the prevalence of inadequate intake in a group requires comparison to the median intake, we also included medians and standard errors (SE) for the nutrients with AI. The percentages of preschoolers with nutrient intakes below or above the recommendations were calculated as previously [32]. The analysis of quantitative variables began with testing the normality using the Shapiro-Wilk statistic with the level of significance set at *P* ≤ 0.05. Statistically significant differences were investigated using the unpaired Student’s t test for normally distributed variables and the non-parametric Mann-Whitney U test for skewed variables with the level of significance set at *P* ≤ 0.05. Qualitative variables were presented in contingency tables. Statistical significance was determined using Pearson’s chi-square test at *P* ≤ 0.05.

## Results

Table 1 shows macronutrient intake in the studied girls and boys from Piła and Table 2 shows the percentages of the studied girls and boys in the reference ranges for macronutrient intake. Boys, in comparison to girls, were found to have statistically significantly higher intakes of total protein (46.7 vs 42.7 g), total protein per kg of body weight, (2.3 vs 2.1 g/kg body weight), saturated fatty acids (24.93 vs 22.51 g), total carbohydrates (220.4 vs 202.5 g), available carbohydrates (209.8 vs 192.4 g), lactose (15.9 vs 14.0 g), sucrose (82.2 vs 70.5 g), and total water (1281 vs 1242 g).

**Table 1:** Macronutrient intake in the studied children from Piła according to gender

Nutrient	Reference values	Girls (*n* = 61)		Boys (*n* = 61)		All children (*n* = 122)		*P*
		Mean	SD	Mean	SD	Mean	SD	
Total protein (g)	body weight dependent	42.7	8.8	46.7	10.3	44.7	9.7	0.022
Total protein (g/kg body weight)	0.84^a^	2.1	0.4	2.3	0.5	2.2	0.5	0.021
Animal protein (g)	NA	28.0	7.8	31.2	8.8	29.6	8.4	NS
Animal protein (% of total protein)	NA	64.9	6.9	66.0	6.6	65.4	6.7	NS
Plant protein (g)	NA	14.7	3.1	15.5	3.2	15.1	3.2	NS
Plant protein (% of total protein)	NA	35.0	6.9	34.0	6.6	34.5	6.7	NS
Total fat (g)	NA	53.6	13.1	57.0	12.2	55.3	12.7	NS
Saturated fatty acids (g)	NA	22.51	5.71	24.93	5.78	23.72	5.85	0.011
Polyunsaturated fatty acids (g)	NA	6.18	1.96	6.28	1.79	6.23	1.87	NS
Monounsaturated fatty acids (g)	NA	21.21	5.93	21.95	5.13	21.58	5.53	NS
Cholesterol (mg)	<300	188	54	198	55	193	54	NS
Total carbohydrates (g)	NA	202.5	36.6	220.4	40.3	211.4	39.3	0.012
Available carbohydrates (g)	130^b^	192.4	34.7	209.8	38.7	201.1	37.6	0.010
Lactose (g)	NA	14.0	8.5	15.9	7.9	14.9	8.2	0.051
Sucrose (g)	NA	70.5	18.3	82.2	23.1	76.3	21.6	0.002
Starch (g)	NA	88.0	19.1	90.3	21.3	89.1	20.2	NS
Dietary fibre (g)	14^c^	10.2 (10.5^d^)	2.7 (0.3^e^)	10.6 (10.1^d^)	2.8 (0.4^e^)	10.4 (10.4^d^)	2.7 (0.2^e^)	NS
Total water (g)	1600^c^	1242 (1227^d^)	223 (29^e^)	1281 (1242^d^)	259 (33^e^)	1262 (1238^d^)	241 (22^e^)	0.048

*P* significance, *NA* not available, *NS* not significant (*P* > 0.05)

^a^EAR, ^b^RDA, ^c^AI, ^d^median, ^e^standard error

**Table 2:** The percentages of the studied children from Piła in the reference ranges for macronutrient intake according to gender

Nutrient	Girls (*n* = 61)	Boys (*n* = 61)	All children (*n* = 122)	*P*
	Percent	Percent	Percent	
Total protein (g/kg body weight)				
Below EAR	0.0	0.0	0.0	#
Cholesterol (mg)				
Within the recommendations	96.7	96.7	96.7	NS
Above the recommendations	3.3	3.3	3.3	
Dietary fibre (g)				
Below AI	96.7	88.5	92.6	NS
AI or above	3.3	11.5	7.4	
Total water (g)				
Below AI	95.1	88.5	91.8	NS
AI or above	4.9	11.5	8.2	

*P* significance, NS not significant (*P* > 0.05)

# – *P* cannot be calculated when percentage is 0.0

Tables 3 and 4 present vitamin intake in the studied girls and boys from Piła and the percentages of the studied girls and boys in the reference ranges for vitamin intake, respectively. Boys, compared to their female peers, were characterised by statistically significantly higher intake of vitamin A (856 vs 702 μg), beta-carotene (2618 vs 2007 μg), vitamin B_2_ (1.582 vs 1.367 mg), vitamin B_12_ (2.61 vs 2.32 μg), and vitamin C (102.0 vs 68.7 mg). Statistically significantly higher percentage of girls (57.4%) than boys (37.7%) had intakes of vitamin E below AI.

**Table 3:** Vitamin intake in the studied children from Piła according to gender

Nutrient	Reference values	Girls (*n* = 61)		Boys (*n* = 61)		All children (*n* = 122)		*P*
		Mean	SD	Mean	SD	Mean	SD	
Vitamin A (retinol equivalent) (μg)	300^a^	702	321	856	390	779	364	0.019
Retinol (μg)	NA	368	187	413	225	390	207	NS
Beta-carotene (μg)	NA	2007	1196	2618	1758	2313	1529	0.042
Vitamin D (μg)	10^a^	2.09	1.43	2.54	2.75	2.31	2.19	NS
Vitamin E (mg)	6^b^	6.24 (5.39^c^)	2.48 (0.32^d^)	6.78 (6.26^c^)	2.78 (0.36^d^)	6.51 (6.02^c^)	2.64 (0.24^d^)	NS
Vitamin B_1_ (mg)	0.5^a^	0.863	0.305	0.972	0.416	0.918	0.367	NS
Vitamin B_2_ (mg)	0.5^a^	1.367	0.453	1.582	0.540	1.475	0.508	0.007
Vitamin B_6_ (mg)	0.5^a^	1.33	0.39	1.46	0.46	1.40	0.43	NS
Folic acid (μg)	160^a^	160.7	37.7	172.4	41.4	166.6	39.9	NS
Vitamin B_12_ (μg)	1.0^a^	2.32	0.91	2.61	0.99	2.46	0.96	0.026
Niacin (mg)	6^a^	9.97	3.18	10.51	3.99	10.24	3.60	NS
Vitamin C (mg)	40^a^	68.7	28.1	102.0	84.3	85.3	64.8	0.035

*NA* not available, *P* significance, *NS* not significant (*P* > 0.05)

^a^EAR, ^b^AI, ^c^median, ^d^standard error

**Table 4:** The percentages of the studied children from Piła in the reference ranges for vitamin intake according to gender

Nutrient	Girls (*n* = 61)	Boys (*n* = 61)	All children (*n* = 122)	*P*
	Percent	Percent	Percent	
Vitamin A (retinol equivalent)				
Below EAR	1.6	0.0	0.8	NS
Retinol				
Above UL	1.6	1.6	1.6	NS
Vitamin D				
Below EAR	100.0	96.7	98.4	NS
Above UL	0.0	0.0	0.0	#
Vitamin E				
Below AI	57.4	37.7	47.5	0.030
Above UL	0.0	0.0	0.0	#
Vitamin B_1_				
Below EAR	3.3	0.0	1.6	NS
Vitamin B_2_				
Below EAR	0.0	0.0	0.0	#
Vitamin B_6_				
Below EAR	0.0	0.0	0.0	#
Above UL	0.0	0.0	0.0	#
Folic acid				
Below EAR	47.5	41.0	44.3	NS
Above UL	0.0	0.0	0.0	#
Vitamin B_12_				
Below EAR	0.0	0.0	0.0	#
Niacin				
Below EAR	6.6	4.9	5.7	NS
Above UL	6.6	8.2	7.4	NS
Vitamin C				
Below EAR	9.8	14.8	12.3	NS
Above UL	0.0	0.0	0.0	#

*P* significance, *NS* not significant (*P* > 0.05)

# – *P* cannot be calculated when percentage is 0.0

Tables 5 and 6 show mineral intake in the studied girls and boys from Piła and the percentages of the studied girls and boys in the reference ranges for mineral intake, respectively. Statistically significantly higher intakes in boys than in girls were observed for calcium (625 vs 529 mg), phosphorus (829 vs 749 mg), and magnesium (179 vs 167 mg).

**Table 5:** Mineral intake in the studied children from Piła according to gender

Nutrient	Reference values	Girls (*n* = 61)		Boys (*n* = 61)		All children (*n* = 122)		*P*
		Mean	SD	Mean	SD	Mean	SD	
Calcium (mg)	800^a^	529	214	625	219	577	221	0.003
Phosphorus (mg)	410^a^	749	169	829	178	789	177	0.012
Magnesium (mg)	110^a^	167	32	179	32	173	32	0.034
Sodium (mg)	1000^b^	2080 (2035^c^)	444 (57^d^)	2223 (2190^c^)	567 (73^d^)	2151 (2121^c^)	512 (46^d^)	NS
Potassium (mg)	3100^b^	1895 (1922^c^)	409 (52^d^)	1974 (1947^c^)	374 (48^d^)	1934 (1941^c^)	393 (36^d^)	NS
Iron (mg)	4^a^	7.1	6.5	6.9	2.2	7.0	4.8	NS
Zinc (mg)	4^a^	5.5	1.2	6.0	1.3	5.8	1.3	NS
Copper (mg)	0.3^a^	0.62	0.13	0.66	0.13	0.64	0.13	NS
Manganese (mg)	1.5^b^	2.39 (2.35^c^)	0.60 (0.08^d^)	2.40 (2.34^c^)	0.50 (0.06^d^)	2.40 (2.35^c^)	0.55 (0.05^d^)	NS
Iodine (μg)	65^a^	77.2	21.2	83.6	28.2	80.4	25.0	NS

*NA* not available, *P* significance, *NS* not significant (*P* > 0.05)

^a^EAR, ^b^AI, ^c^median, ^d^standard error

**Table 6:** The percentages of the studied children from Piła in the reference ranges for mineral intake according to gender

Nutrient	Girls (*n* = 61)	Boys (*n* = 61)	All children (*n* = 122)	*P*
	Percent	Percent	Percent	
Calcium				
Below EAR	88.5	82.0	85.2	NS
Above UL	0.0	0.0	0.0	#
Phosphorus				
Below EAR	1.6	0.0	0.8	NS
Above UL	0.0	0.0	0.0	#
Magnesium				
Below EAR	4.9	0.0	2.5	NS
Sodium				
Below AI	0.0	0.0	0.0	#
Above UL	90.2	95.1	92.6	NS
Potassium				
Below AI	100.0	100.0	100.0	#
Iron				
Below EAR	4.9	0.0	2.5	NS
Above UL	1.6	0.0	0.8	NS
Zinc				
Below EAR	8.2	4.9	6.6	NS
Above UL	0.0	0.0	0.0	#
Copper				
Below EAR	0.0	0.0	0.0	#
Above UL	0.0	0.0	0.0	#
Manganese				
Below AI	4.9	4.9	4.9	NS
Above UL	11.5	13.1	12.3	NS
Iodine				
Below EAR	27.9	27.9	27.9	NS
Above UL	0.0	0.0	0.0	#

*P* significance, *NS* not significant (*P* > 0.05)

# – *P* cannot be calculated when percentage is 0.0 or 100.0

## Discussion

### General comments

In the previous article [33], we presented sociodemographic characteristics and selected indices of health status of the studied preschoolers and their families. However, in the current article we did not present nutrient intake of six children, that is two girls and four boys, because their parents provided incomplete food records. Nevertheless, it does not change the overall characteristics of this population. Although the preschools for the intervention were selected randomly, the studied children’s parents turned out to be better educated than their peers in the general population [33]. Moreover, high percentages of them assessed the economic status of the family as good or very good [33]. These findings, together with the fact that the parents voluntarily gave their informed consent to take part in the study, would suggest an expectation that the parents would be interested in providing a healthy diet to their children. Additionally, the high percentages of the studied children’s parents who reported diet-related diseases in their families [33] are another factor which should induce parents to follow current nutrition recommendations, irrespective of their child’s gender.

In the previous article [23], we also presented a detailed analysis of energy intake in relation to the studied children’s BMI. These results indicated a low probability of misreporting children’s food intake since energy intake increased through the percentile categories for BMI unlike energy intake in other groups of children in the same or similar age range [34–38].

### Gender differences

In the studied preschool children from Piła, gender turned out to be a significant factor of nutrient intake, since intakes of as many as 16 nutrients out of 40 were statistically significantly different in girls and boys. However, in two previously published studies on nutrient intake in preschool children, intake of even more nutrients differed according to gender, that is 24 nutrients in 4-year-old urban children from all over Poland [39] and 17 nutrients in Belgian 4–6.5-year-old preschoolers [40]. In the remaining studies, in which the influence of gender on nutrient intake in preschoolers was analysed, less differences were found [32, 37, 41–46].

Among the nutrients on which gender had statistically significant influence, seven nutrients in the group of 4-year-old urban children from all over Poland [39] were the same as in the studied preschoolers. These nutrients included: total protein, saturated fatty acids, available carbohydrates, vitamin A, vitamin B_12_, phosphorus and magnesium. In Belgian 4–6.5-year-old preschoolers [40], eight nutrients on which gender had statistically significant influence were the same as in the studied preschoolers, that is: total protein, total carbohydrates, total water, vitamin B_2_, vitamin C, calcium, phosphorus and magnesium.

It is interesting to note that the nutrients which differed most often according to gender were total protein and magnesium: statistically significant differences were found in the studied preschoolers, in urban 4-year-olds from all over Poland [39], and in Belgian 4–6.5-year-olds [40].

It is noteworthy that intakes of all the nutrients on which gender had statistically significant influence, and also intakes of all the remaining nutrients except for iron, were higher in the studied boys compared to girls. It is natural due to the higher energy intake among boys found in the studied children [23] and in other preschoolers [37–40, 46].

### Assessment of macronutrient intake

The favourable feature of the studied preschoolers’ macronutrient intake was adequate intake of monounsaturated fatty acids and cholesterol. Intake of these macronutrients was also adequate in other Polish preschoolers [37, 39, 42, 46–48], as well as in preschool children from other countries [40, 49–53]. Adequate intake of monounsaturated fatty acids in Polish children results from the popularity of rapeseed oil which in Poland is produced in large amounts [54]. In Belgium, Finland and Great Britain rapeseed oil production is quite high and the highest of all other oils [54], whereas Spain and Greece are known for the widespread use of olive oil [55]. It is also not surprising that cholesterol intake falls under the upper limit recommended by the World Health Organization (WHO) in the prevention of diet-related diseases [31] since energy intake at this stage of life is relatively low in comparison to energy intake of adults and the limit is the same irrespective of age. However, those children whose cholesterol intake was close to this limit [37, 46, 50] are at risk of excessive cholesterol intake in the future because the increase of energy intake, which must accompany the increase of body height and body mass, may result in the increased intake of cholesterol. This may be observed in Spanish children [50]: 2–5-year-olds consumed slightly less cholesterol than the upper limit, while 6–9-year-olds exceeded the WHO recommendations. Of course, one may claim that the amount of cholesterol in the diet is not important because recent recommendations of the European Food Safety Authority [56] and the Dietary Guidelines Advisory Committee of the United States Department of Agriculture [57] have not included any limitations of cholesterol intake. However, in our opinion this claim is not adequate for two reasons. First of all, the fact of not including the limits on cholesterol intake in the dietary guidelines worked out by both European and American experts [56, 57], does not mean that cholesterol may be consumed in large amounts, as was interpreted by mass media. The experts did not state that high cholesterol intake is good for cardiovascular health. They only stated that the evidence is not adequate enough for setting a quantitative limit. Besides high-cholesterol foods are usually rich source of saturated fatty acids and these fatty acids should be limited in the daily diet [58]. Moreover, examples of healthy eating patterns in the American guidelines in fact limit dietary cholesterol to a range of 100 to 300 mg/day [58]. The second reason is the high prevalence of cardiovascular diseases in the studied children’s families reported previously [33] which indicates the necessity to pay special attention to nutritional factors which are able to prevent the onset of the diseases in genetically predisposed children. Therefore, it was highly favourable that cholesterol content in the studied children’s diets did not exceed the WHO recommendations [31].

Protein intake both in the studied preschool children and in other Polish preschoolers was either adequate [37, 39, 46–48] or excessive [42], similar to children from other countries who consumed either adequate [40, 49, 51–53] or excessive [41, 50] amounts of this macronutrient. Such results are not surprising because nowadays inadequate protein intake resulting in malnutrition at the community level is observed only in low-income countries.

Another features of the studied preschoolers’ diets which were common to the diets of both children from Poland and children from other countries were excessive intake of total fat [37, 39, 41, 47–53], excessive intake of saturated fatty acids [37, 39–42, 47, 49–53], inadequate intake of polyunsaturated fatty acids [37, 39, 40, 42, 47, 50–53] and excessive intake of sucrose, simple carbohydrates or non-milk extrinsic sugars [37, 39, 40, 42, 48, 51, 53]. Such low intake of dietary fibre as in the studied preschoolers was observed only in one study on Polish children [42] and in three studies on children from other countries [50, 51, 53]. These characteristics of the children’s diets increase the risk of diet-related diseases, especially atherosclerosis [59–62]. It is particularly unfavourable when taking into account that ischaemic heart disease and stroke are the two main causes of death in Europe [63] and that the prevalence of familial myocardial infarction in the studied population was relatively high [33]. Moreover, these observations, irrespective of the country, indicate that intervention is necessary to prevent future health consequences, particularly taking into account the trends in food intake. These trends show that the frequency of consuming meat products, sweets and sugar-sweetened carbonated beverages among children increases with age [64, 65], while the frequency of consuming fruit and vegetables decreases with age [64]. Also, energy-adjusted intake of sweets and sugar increases with children’s age, while energy-adjusted intake of vegetables and fruit decreases with age [51].

Another unfavourable feature of the studied preschoolers’ diets was inadequate total water intake. Intake of this macronutrient has been rarely included in nutrient intake assessment in preschool children. It was analysed only in two studies on Polish preschoolers [37, 42] and one study on children from Belgium [40]. All of these studies reported water intake to be inadequate. Due to the role of water in preventing chronic diseases [66], more research on water intake in preschoolers should be carried out. Preschool children should be encouraged to drink more water and preschool staff should be educated about the need to promote water intake in children.

### Assessment of micronutrient intake

Intake of most vitamins and minerals was adequate in the studied preschool children, that is intake of vitamin A, B_1_, B_2_, B_6_, B_12_, PP, C, phosphorus, magnesium, iron, zinc, copper, manganese and iodine. Intake of these micronutrients was also reported to be adequate in Polish preschoolers studied previously [32, 39, 43–48] as well as in preschool children from other countries [40, 49–51, 53]. However, there were some serious nutrition concerns which included inadequate intake of vitamin D, E, folic acid, calcium and potassium, along with excessive intake of sodium.

The most serious nutritional concern in the studied preschoolers’ diets was inadequate intake of vitamin D observed in all of the studied girls and almost all of the studied boys. Such low intakes of vitamin D are typical of preschool children both in Poland [32, 39, 43, 46] and other countries [40, 50, 51, 53]. Unfortunately, this finding confirms the prognosis that osteoporosis incidence in Europe will rise [67] and unless intervention programmes will be introduced to prevent inadequate intakes of vitamin D in children, osteoporosis morbidity will remain on the rise. Moreover, since vitamin D was reported as a caries-preventive agent [68], inadequate intake of this vitamin, together with the abovementioned excessive sucrose intake, may increase the prevalence of dental caries among the studied preschoolers. Of course, apart from dietary sources, vitamin D is also synthesised in the skin during exposure to ultraviolet radiation [69]. Nevertheless, total annual sunshine in Poland is low and does not exceed 1800 h [70]. For comparison, in Spain and Greece total annual sunshine exceeds 2500 h [70]. Therefore, it is not possible that the serious dietary shortcomings in vitamin D observed in the studied children would be compensated by cutaneous synthesis.

Another serious nutrition concern in the studied preschoolers was high prevalence of inadequate calcium intake along with high risk of inadequate potassium intake and excessive sodium intake similar to other Polish children studied previously [32, 39, 45, 47] and British preschoolers [49]. Two studies on Polish children [46, 48] reported adequate sodium intake but confirmed inadequate intake of calcium and potassium, whereas studies on Belgian [40] and Spanish [50] children reported adequate calcium intake but confirmed inadequate potassium intake and excessive sodium intake. These findings require urgent intervention because inadequate intake of calcium and potassium together with excessive intake of sodium not only increases the risk of hypertension but also increases the risk of osteoporosis, which is additionally aggravated by the abovementioned inadequate intake of vitamin D [71].

Although mean intakes of vitamin E and folic acid were above AI and EAR, respectively, it is highly disconcerting that the prevalence of inadequate intake of these vitamins in the studied preschool children was quite high. Most of the previously published studies on Polish preschoolers also reported that mean intakes of vitamin E [32, 39, 47, 48] and folic acid [32, 39, 46] were higher than AI and EAR, respectively. The prevalence of inadequate intake was analysed only in three Polish studies [32, 43, 44]: two studies confirmed the high prevalence of inadequate intake of vitamin E [32, 43] and one study confirmed the high prevalence of inadequate folic acid intake [44]. Among the few studies which reported vitamin E and folic acid intake in preschool children from other countries, the results were varied: some studies indicated inadequate intake of vitamin E [50, 51] and folic acid [50, 51], while other showed adequate intake of vitamin E [49, 53] and folic acid [53]. The insufficient intake of vitamin E and folic acid observed in the studied preschoolers and in several other groups of children shows the need to target these nutrients in special nutrition intervention programmes addressed to preschool children and their parents. It is important since inadequate intake of these vitamins poses a health hazard in the context of preventing diet-related diseases. Vitamin E was recognised to have anticancer potential [72] and was found to reduce two risk factors of cardiovascular diseases, that is hypertension and high waist circumference [73]. Vitamin E is also known to inhibit oxidative modification of LDL lipoproteins and may inhibit atherogenesis through several other mechanisms at the molecular and cellular levels [74]. Folic acid was found to be inversely associated with risk of coronary heart disease [75]. Taking into account the crucial role of these vitamins in preventing diet-related diseases, more studies should include assessment of vitamin E and folic acid intake in preschool children in order to prevent any possible deficiencies in this young population.

It is also worth noting that although mean iodine intake was higher than EAR, the prevalence of inadequate iodine intake may also be a matter of concern: almost 1/3 of the studied preschoolers had intakes of this mineral lower than EAR. Iodine intake in preschool children was assessed only in two other Polish studies [32, 45] and one study on British children [53]. All of these studies reported adequate iodine intakes. However, it should be noted that in fact it is difficult to assess iodine intake based on food records and food composition tables because iodine content in foods may vary significantly, for example iodine content in milk depends on the amount of iodine consumed by the animal [26], iodine content in plants depends on the amount of iodine in the soil which may vary in different regions of the country, etc. Therefore, these results show the need to assess iodine status of the studied children. The confirmation are the results of the study in preschoolers from Nowy Scz and the vicinity [32] whose iodine intake assessed from food records was adequate, but not even a half of them was characterised by adequate iodine status as implied by urinary iodine concentration [76].

Another disconcerting feature of the studied preschoolers diets may be the high prevalence of excessive manganese intake. Similar [45] or even higher [32] prevalence was observed also in other Polish preschoolers. The remaining few studies which reported intake of this mineral in preschool children included only mean intake [39, 47, 49] and showed higher mean manganese intake in other Polish children [39, 47] but lower intake in British children [49]. The children whose manganese intake exceeds UL should have blood manganese concentration measured to assess whether their intake of this mineral is in fact too high, because absorption of dietary manganese is relatively low, depends on the source of manganese being higher from water and supplements than from foods, and is affected by several dietary factors [26], such as lowering effect of phytate on manganese absorption [77].

### Study strengths and limitations

Our study showed that gender was a significant factor of nutrient intake in preschool children and that the studied preschoolers’ diets were characterised by excessive intakes of saturated fatty acids, sucrose and sodium along with inadequate intakes of polyunsaturated fatty acids, dietary fibre, total water, vitamin D, vitamin E, folic acid, calcium and potassium. All of these concerns were similar to the previously published results on preschool children from various countries. Also, a matter of concern may be those children who exceeded UL for manganese and those who had inadequate iodine intakes, which in the case of these two minerals requires blood manganese concentration assessment and urinary iodine concentration assessment, respectively.

When considering these findings, the strengths and limitations of our study should be taken into account. The strengths are (1) a seven-day period of keeping food records, (2) using electronic scales to weigh all foods and beverages, (3) including water and dietary supplements, (4) keeping individual records also during preschool hours.

Our study has also some limitations. Despite the fact that the authors did their best to motivate parents and preschool staff to keep the food records as precisely as possible, it was not feasible to supervise each preschool teacher constantly and to assist parents to check if their records kept out of preschool were precise indeed. Although energy intake in the studied preschool children increased through the percentile categories for BMI as reported previously [23], which suggested low risk of underreporting, and although the food records included in the analysis seemed to be filled in carefully, underreporting cannot be excluded even if the results are within the plausible range. However, this limitation refers to all studies on dietary assessment and the only thing the authors might do is to minimise the risk of imprecision.

## Conclusions

In conclusion, gender was a significant factor of nutrient intake in the studied preschool children. The main nutritional concerns in the studied preschoolers’ diets, irrespective of gender, are typical of the diets of preschool children from various parts of Europe and pose the risk of developing diet-related diseases in adulthood, especially atherosclerosis, hypertension and osteoporosis. These findings indicate the need to work out common nutritional strategies to improve preschoolers’ nutrition across Europe which will result in the reduction of future social and economic burden of diet-related diseases to the European societies. Moreover, it is necessary to carry out more research on intake of water, manganese and iodine in preschool children and to carry out research on children’s nutritional status assessed from blood indices.

## Abbreviations

AI: Adequate Intake
DRVs: Dietary Reference Values
EAR: Estimated Average Requirement
RDA: Recommended Dietary Allowance
UL: Tolerable Upper Intake Level
